# Tolerance to Self: Which Cells Kill

**DOI:** 10.1371/journal.pbio.0060241

**Published:** 2008-09-30

**Authors:** Terri M Laufer

## Abstract

Immune self tolerance involves the deletion in the thymus of developing T cells that have the ability to recognize self-antigens, but by which cells? New evidence argues that cortical epithelial cells can induce deletion of self-reactive T cells.

The mammalian immune system is tightly controlled to be activated by infectious agents but not by self. The ability to respond to the universe of potential antigens is mediated by a repertoire of T and B lymphocytes with an extraordinary range of specificities. The diversity of T and B lymphocyte antigen receptors is generated by completely random rearrangement of gene segments. Thus, this diverse repertoire must be purged of autoreactive specificities—a process known as negative selection. T cell tolerance is established in the thymus, an organ uniquely specialized to support the development of T cells and regulate their self-tolerance. In a recent paper published in *PLoS Biology* [1], Ahn and colleagues delve into a longstanding question about the process of negative selection: which cells in the thymus induce tolerance to self to prevent autoimmunity?

The developmental fate of thymocytes, T cell precursors, is regulated by signals delivered to the T cell receptor (expressed on the thymocyte) by peptide/major histocompatibility complex (MHC) molecules expressed on thymic stromal cells. Developing thymocytes interact with peptide/MHC molecules expressed by both thymic epithelial cells and bone marrow–derived dendritic cells (DCs). The thymus is divided into two distinct regions: an outer cortex and an inner medulla. Thymic epithelial cells are present in both regions; DCs primarily localize to the medulla and the corticomedullary junction. Cortical and medullary epithelial cells arise from a single precursor but are phenotypically and functionally quite distinct, and, as shown by the current authors and others, exist as heterogeneous populations.

T cell development occurs in specialized thymic microenvironments established by the stromal cells. For many of these processes, the localization of the event and the thymic cell mediating that process are quite clear. T cell precursors enter the thymus at the corticomedullary junction and rearrange T cell receptor (TCR) genes in the cortex. Immature thymocytes in the cortex integrate primary signals transduced by the TCR with secondary, costimulatory signals. If an inadequate signal is received, the cell commits suicide: death by neglect. Positive selection is the crucial step in which thymocytes that can interact productively with self-peptide/MHC molecules survive and differentiate further. Immature thymocytes screened for positive selection localize to the outer cortex, and it is well accepted that cortical epithelium is uniquely capable of directing the positive selection of traditional aß-expressing T cells. The process of rescuing cells that can interact with self-peptide/MHC complexes enriches for autoreactive T cells. Thus, tolerance mechanisms must be in place to prevent autoimmune disease in the mature organism. The hallmark of negative selection is the clonal deletion of thymocytes with high affinity for self-antigens (reviewed in [2]). Despite years of investigation, the timing and regulation of clonal deletion remain incompletely understood.

Multiple models for negative selection have been proposed. One hypothesis is that fate decisions are determined by the strength of the integrated signal received by the developing thymocyte; T cell progenitors with high affinity for self-peptide/MHC will be killed. In this model, high-affinity interactions between the progenitor and any thymic stromal cell will lead to clonal deletion. A second hypothesis proposed by us and others argues that positive and negative selection events are anatomically separated [3]. In this model, positive selection in the thymic cortex precedes negative selection in the thymic medulla. In this distinct model, DCs and medullary epithelial cells in the thymic medulla would interact with semi-mature thymocytes and induce most clonal deletion. In the setting of this second hypothesis, the ability of thymic cortical epithelium to mediate clonal deletion remains controversial.

This latter model makes intuitive sense. There are no significant immunologic consequences if an immature thymocyte fails to find a positively selecting peptide/MHC complex. In contrast, the failure to negatively select even one autoreactive T cell could be catastrophic for the organism. To avoid autoimmunity, the developing thymocyte must scan the entire universe of self-peptide/MHC complexes. It simplifies this task to limit negative selection to only relevant thymocytes—that very small percentage of thymocytes that have already been positively selected. Thus, a plan in which cells are first positively selected in the thymic cortex and then migrate to the medulla to undergo screening for autoreactivity is appealing.

The current authors have examined the ability of a subpopulation of thymic cortical epithelial cells to mediate clonal deletion. This group previously identified a very small subset of cortical epithelial cells—about 4,000 cells per murine thymus—that express thymic stromal cotransporter (TSCOT), a putative 12-transmembrane protein [4,5]. In the new study, the group took advantage of a novel genetically modified mouse that expresses a small amount of the bacterial protein, LacZ (a few thousand molecules/cell), in TSCOT-positive cells. LacZ functions as a reporter protein, which the authors were able to use to easily identify TSCOT-positive cells. However, because LacZ is continuously expressed in thymic epithelial cells, it is also a well-defined self-antigen to which developing thymocytes are exposed. This technique allowed Ahn et al. to determine if T cells were tolerant to this new self-antigen.

Interestingly, the T cell compartment is tolerant of LacZ; TSCOT/LacZ-transgenic mice do not mount an immune response to LacZ following immunization with exogenous LacZ. Thus, cortical epithelium can induce tolerance to endogenous proteins. But what is the mechanism of this tolerance? Previous work has clearly shown that cortical epithelial cells can mediate tolerance. Previous results suggested that thymic epithelium tolerized T cells by inducing anergy, a state in which the T cell fails to respond to its cognate antigen [6,7]. Separately, we and others have shown that cortical epithelium directs the development of a special class of T cells, regulatory T cells, which prevent autoimmunity by suppressing the activation of autoreactive T cells [8]. But can cortical epithelium mediate clonal deletion? Ahn et al. examine this more controversial hypothesis.

To determine if tolerance was induced by clonal deletion, the authors used the standard immunologic technique of examining T cell development in mice with a T cell repertoire in which almost all thymocytes express a LacZ-specific TCR. They found a complete absence of LacZ-specific thymocytes—both mature and immature—and concluded that clonal deletion had occurred. The authors additionally examined clonal deletion in artificial re-aggregated thymi. This allowed them to demonstrate that antigen presentation by LacZ-positive thymic cortical epithelial cells was sufficient to prevent the development of mature single positive CD4^+^ T cells; neither thymic medullary epithelial cells nor DCs were necessary. Thus, the authors conclude that TSCOT-positive cortical epithelial cells mediate clonal deletion of autoreactive CD4^+^ T cells—this ability is not limited to medullary epithelial and DCs.

Do these findings alter current models of negative selection? Certainly, they are in agreement with previous papers, including one published by the current authors [9] suggesting that thymic cortical epithelial cells can induce deletion of self-reactive T cells. However, there are caveats to these interpretations. One centers on details about the LacZ-specific TCR transgenic mouse used in the current study. The analysis of thymic development in the transgenic TSCOT mice shows an absence of immature preselection thymocytes in the presence of self-antigen, a phenotype seen in other TCR transgenic systems in which the TCR is expressed inappropriately early. In these cases, the absence of immature thymocytes results from a block in differentiation rather than from clonal deletion and is probably artifactual [10]. Thus, it will be important to carefully examine the expression of the anti-LacZ TCR once those data become available.

A juvenile mouse thymus contains approximately 4–5 × 10^5^ epithelial cells [11]. So it's quite striking that TSCOT-positive cortical epithelial cells can tolerize the entire T cell repertoire, even when that repertoire is vastly skewed toward self-reactivity. The authors have done a lovely—and important—accounting job: around 4,000 thymic epithelial cells, each expressing a few thousand LacZ molecules, can mediate the deletion of a repertoire completely skewed toward autoreactivity. How might this work? TSCOT-positive thymic epithelial cells express costimulatory molecules such as CD80 and CD54 that provide necessary second signals to thymocytes for clonal deletion [12]. This agrees with what we know about necessary molecular requirements for deletion. Thus, TSCOT-positive cells are phenotypically similar to the medullary epithelial cells and DCs that mediate negative selection, rather than the majority of cortical epithelial cells.

The current observations may conflict with models suggesting that clonal deletion only occurs late in thymic development during medullary residency. Thus, it will be important to determine when thymocytes interact with TSCOT-positive cells. Is negative selection sequential—do thymocytes interact with TSCOT-positive cells after they have received a positive selection signal—or can deletion of immature thymocytes that have not yet been positively selected occur, as has been suggested by unmodified affinity models of selection? To answer these questions, it will be very interesting to define the timing of TSCOT-mediated deletion by examining expression of apoptotic proteins such as Nur77 induced during clonal deletion [13]. Similarly, we look forward to seeing in vivo imaging of developing thymocytes interacting with TSCOT-positive thymic epithelial cells—how can so few thymic cortical epithelial cells efficiently delete an entire repertoire? These studies should permit a more nuanced understanding of the complicated processes of thymic selection.

## Abbreviations

DC: dendritic cell
MHC: major histocompatibility complex
TCR: T cell receptor
TSCOT: thymic stromal cotransporter
